# Identification of a lactylation-related gene signature in microsatellite stable gastric cancer based on bulk and single-cell RNA-seq

**DOI:** 10.3389/fimmu.2026.1838771

**Published:** 2026-05-21

**Authors:** Zhong-Tai Lin, Yuan-Chun Chen, Li-Sheng You, Yuan-Zhao Wang, Xiang-Yu Wang, Liang-Jie Chi

**Affiliations:** 1 The Shengli Clinical Medical College of Fujian Medical University, Fujian Provincial Hospital, Fuzhou, Fujian, China; 2 Department of Gastrointestinal Surgery, Fuzhou University Affiliated Provincial Hospital, Fuzhou, Fujian, China; 3 Department of Gastroenterology, Fuzhou University Affiliated Provincial Hospital, Fuzhou, Fujian, China; 4 Department of Pathology, Fuzhou University Affiliated Provincial Hospital, Fuzhou, Fujian, China

**Keywords:** gastric cancer, immunotherapy, lactylation-related genes, microsatellite stable, single-cell analysis, tumor microenvironment

## Abstract

**Background:**

Microsatellite stable (MSS) gastric cancer (GC) responds poorly to immunotherapy and exhibits heterogeneous outcomes. Histone lactylation plays a critical role in cancer progression. However, the prognostic and therapeutic potential of a lactylation-related gene signature (LRGS) in MSS GC remains largely unexplored.

**Methods:**

Data of MSS GC patients were obtained from The Cancer Genome Atlas and Gene Expression Omnibus databases. Consensus clustering based on lactylation-related gene expression profiles was performed to stratify patients. We further constructed the LRGS using machine learning algorithms. We also assessed its correlations with clinicopathological parameters, the tumor microenvironment, and chemosensitivity. The Tumor Immune Dysfunction and Exclusion (TIDE) algorithm was employed to predict potential responses to immunotherapy. Single-cell analysis, cell-cell communication analysis, in silico knockout, and immunohistochemical validation were integrated to explore the functional roles of key genes.

**Results:**

Consensus clustering uncovered two clusters with significantly different overall survival outcomes. A nine-gene LRGS was established and effectively stratified patients into high- and low-risk groups. The high-risk group displayed an immunosuppressive microenvironment, reduced chemosensitivity, and higher TIDE scores. Single-cell analysis revealed that LRGS scores were highest in cancer-associated fibroblasts (CAFs), with *APOD* and *SERPINE1* highly expressed in PDGFRA+ CAFs and CA9+ CAFs, respectively. Cell-cell communication analysis showed that these two CAF subtypes exhibited distinct signaling patterns via the COLLAGEN and MIF pathways. In silico knockout further validated that *APOD* and *SERPINE1* are essential for matrix remodeling and hypoxia adaptation in their respective CAF subtypes. Immunohistochemical analysis confirmed elevated protein levels of *APOD* and *SERPINE1* in MSS GC.

**Conclusions:**

This study developed and validated a robust LRGS for MSS GC. This signature facilitates accurate prognosis prediction and shows potential to predict responses to both chemotherapy and immunotherapy.

## 1 Introduction

Gastric cancer (GC) is one of the most common malignant tumors worldwide, with persistently high incidence and mortality rates posing a serious threat to public health (1). Although certain progress has been made in recent years in treatment approaches including surgery, chemotherapy, targeted therapy, and immunotherapy (2–5), the survival outcomes of GC patients remain poor, with a 5-year survival rate below 30% (6, 7). GC exhibits high heterogeneity and its development involves multiple factors, including genetic variants, metabolic abnormalities, and complex regulation of the tumor microenvironment (TME) (8–11).

Microsatellite instability (MSI) is an important molecular characteristic of GC, typically resulting from dysfunction of the DNA mismatch repair system (12). Studies have shown that patients with MSI-high GC exhibit higher response rates to immunotherapy, whereas those with microsatellite stable (MSS) GC respond poorly to immunotherapy (13–15). As the predominant subtype of GC, MSS GC is characterized by an immunosuppressive TME, manifesting as inadequate immune cell infiltration and immune escape (9, 16, 17). These features contribute to limited treatment options and a generally poorer prognosis (18, 19). Therefore, in-depth investigation into the molecular mechanisms of MSS GC and the development of biomarkers capable of predicting prognosis and immunotherapy response are of great importance for guiding individualized treatment.

Recently, the role of lactate metabolism in the regulation of the TME has garnered increasing attention. Lactate is not merely an end product of glycolysis but also participates in tumor proliferation, metastasis, and immune regulation through epigenetic modification mechanisms such as histone lactylation (20). A recent study has revealed that *AARS1* functions as a lactyltransferase catalyzing *YAP*–*TEAD* lactylation, thereby promoting GC proliferation (21). Additionally, histone lactylation has been reported to upregulate *METTL3* expression, facilitating tumor proliferation while suppressing CD8+ T cell cytotoxicity (22). It also promotes *FAP* transcription and extracellular matrix remodeling, contributing to CD8+ T cell exclusion and immunosuppressive niche formation in GC (23). This suggests that lactylation may inform prognosis and immunotherapy response in MSS GC. To explore this, we constructed a lactylation-related gene signature (LRGS) and systematically characterized its heterogeneity across key dimensions, including the TME, chemotherapy sensitivity, and immunotherapy sensitivity, with validation of its cellular basis at single-cell resolution. Given the critical role of intercellular communication in tumor progression (24, 25), we performed cell-cell communication analysis. Additionally, in silico knockout (KO) analysis was used to predict the functional roles of key genes. This LRGS is poised to support molecular subtyping and individualized treatment strategies for MSS GC.

## 2 Materials and methods

### 2.1 Data acquisition and processing

The stomach adenocarcinoma (STAD) dataset was downloaded from The Cancer Genome Atlas (TCGA), which contained transcriptome data of 448 patients with STAD, including 412 tumor and 36 normal samples. The MSI or MSS status of the TCGA-STAD samples was obtained from the cBioPortal database. Besides the transcriptome data, clinical information such as age, gender, TNM stage, and survival status was also included in this cohort. Following the exclusion of normal samples, MSI samples, and samples without survival data, a final cohort of 314 MSS GC tumor samples was analyzed. The Gene Expression Omnibus (GEO) dataset GSE62254 served as an external validation dataset to assess the robustness of the signature. The lactylation-related genes were obtained from previous studies (26–28) and are listed in **Supplementary Table 1**.

### 2.2 Identification of lactylation-related clusters in MSS GC

To identify potential clusters of STAD based on lactylation-related genes, we performed a consensus clustering analysis. We first performed univariate Cox regression analysis to identify genes significantly associated with overall survival (OS) for subsequent analysis. Then, unsupervised consensus clustering was performed on these significant genes using the R package “ConsensusClusterPlus”. The analysis was configured with a maximum cluster number of 9 and 1000 resampling iterations to evaluate the stability of the clustering and determine the optimal K value. To evaluate the prognostic differences between clusters, we conducted survival analysis to compare OS between the clusters using the R package “survminer” and plotted Kaplan–Meier survival curves.

We analyzed the associations of the clusters with key clinical parameters, including age, gender, and TNM stage. To characterize the TME, we applied the CIBERSORT algorithm to quantify the infiltration levels of 22 immune cell types. Additionally, the ESTIMATE algorithm was used to compute ImmuneScore, StromalScore, and ESTIMATEScore, providing a comprehensive profile of the TME composition in each sample.

### 2.3 Construction and validation of the LRGS

To identify differentially expressed genes (DEGs) between the clusters, we performed an analysis of the RNA-seq data using the “DESeq2” R package. DEGs were defined as those with an absolute |log2FC| > 1 and an adjusted p-value < 0.05. Subsequently, Gene Ontology and Kyoto Encyclopedia of Genes and Genomes pathway enrichment analyses were conducted on these DEGs using the “clusterProfiler” R package. The significance of the enrichment results was determined with a cut-off criterion of a p-value < 0.05 and a false discovery rate < 0.05 to identify significantly enriched biological functions and signaling pathways between the clusters.

We used Least Absolute Shrinkage and Selection Operator (LASSO) regression to reduce dimensionality among prognostic DEGs, identifying 15 candidate genes. Subsequently, we integrated ten machine learning algorithms—Partial Least Squares Regression for Cox (plsRcox), Random Survival Forest (RSF), CoxBoost, Elastic Net (Enet), Gradient Boosting Machine (GBM), Survival Support Vector Machine (survival-SVM), Supervised Principal Component (SuperPC), StepCox, Ridge, and LASSO—to generate 101 algorithm combinations. All models were implemented using the “Mime1” R package and evaluated by the concordance index (C-index) in both the training and validation datasets. Based on the ranking of mean C-index, the model with the highest mean C-index was selected to construct the LRGS. Patients were stratified into high- and low-risk groups using the median risk score as the cutoff. The associations among consensus clusters, risk score, and survival status were visualized using the “ggpubr” and “reshape2” R packages.

### 2.4 Analysis of TME based on LRGS

To explore the association between the LRGS and immune cell infiltration, we quantified the abundances of 22 immune cell types using the CIBERSORT algorithm. Spearman correlation analysis was then performed to evaluate the relationship between the LRGS score and immune cell abundance. Furthermore, the expression levels of key immune checkpoint-related genes were compared between the high- and low-risk groups.

### 2.5 Immunotherapy and chemotherapy response prediction based on LRGS

To assess the drug sensitivity of each sample, we applied the “oncoPredict” R package to predict the half-maximal inhibitory concentration (IC50) of commonly used chemotherapy for GC patients. This method utilizes expression data and drug sensitivity models derived from the Genomics of Drug Sensitivity in Cancer database. Subsequently, the Tumor Immune Dysfunction and Exclusion (TIDE) algorithm was employed to evaluate the potential immunotherapy resistance of each sample based on its gene expression profile.

### 2.6 Single-cell RNA sequencing analysis

Single-cell RNA sequencing data were downloaded from the GEO database (accession: GSE183904) and analyzed using the R package “Seurat” (version 5.4.0). Stringent quality control criteria were implemented: cells expressing fewer than 200 genes or more than 6000 genes, as well as cells with mitochondrial gene percentage exceeding 10%, were excluded. Data were log-normalized using the “NormalizeData” function. The top 3000 highly variable genes were selected using the “FindVariableFeatures” function for principal component analysis-based dimensionality reduction. The “harmony” package was applied to remove batch effects across samples. We visualized the identified clusters using Uniform Manifold Approximation and Projection. Cell clusters were annotated based on typical markers. The “AddModuleScore” function was used to calculate the module score for each cell based on the LRGS genes. Differential expression analysis was conducted using the “FindMarkers” function with the Wilcoxon rank-sum test, with thresholds set at |log_2_FC| > 0.25 and adjusted p-value < 0.05.

### 2.7 Cell-cell communication analysis

We employed “CellChat” to investigate the molecular interaction networks among different cell types. This analysis inferred intercellular communication probabilities and predicted relevant signaling pathways based on the law of mass action and the expression levels of ligand-receptor pairs. Furthermore, pattern recognition was applied to analyze the global communication features of cell subsets, and in-depth network construction and functional role quantification were performed for key pathways. The results were visualized using circle plots, network diagrams, heatmaps, and other formats to display the number, strength, and patterns of communication. The activity of each pathway was compared to assess its relative contribution.

### 2.8 In silico KO analysis of key genes

To investigate the role of key genes, we performed in silico KO analysis using the “scTenifoldKnk” R package. This method constructs gene regulatory networks from single-cell transcriptomic data and identifies downstream DEGs by simulating the KO state. Functional enrichment analysis was subsequently performed on the significant DEGs to elucidate the associated biological processes and pathways.

### 2.9 Immunohistochemistry (IHC) staining and scoring

A total of 10 paired GC and adjacent normal tissue specimens were collected for IHC validation between January 2025 and May 2025. All specimens were obtained from postoperative patients with MSS GC treated at our hospital. The specimen collection protocol was approved by the Ethics Committee of our hospital (Approval No. K2026-05-021), and informed consent was obtained from all patients. GC tissue paraffin specimens were cut into 4 μm thick serial sections, dewaxed, and hydrated. Antigen retrieval was performed in sodium citrate antigen retrieval solution (pH 9.0). Endogenous peroxidase blocker was applied and incubated at room temperature for 30 min, followed by phosphate-buffered saline (PBS) washing. Sections were then incubated with *APOD* antibody (Proteintech, 10520-1-AP, 1:400) and *SERPINE1* antibody (Proteintech, 13801-1-AP, 1:400) at 37 °C for 1 h. After PBS washing, signal amplification agent and enzyme-labeled polymer were sequentially applied, each incubated at room temperature for 15 min with PBS washing after each incubation. 3,3’-diaminobenzidine substrate was used for color development for 2 min, then washed. Hematoxylin was applied for 10–30 s, and sections were rinsed with tap water for blueing. Finally, sections were dehydrated, cleared, and mounted.

The staining results of *APOD* and *SERPINE1* were independently evaluated by two experienced pathologists at Fuzhou University Affiliated Provincial Hospital. Staining intensity was scored as: 0 (negative), 1 (weak), 2 (moderate), and 3 (strong). The proportion of positive cells was scored as: 0 (0–5%), 1 (6–25%), 2 (26–50%), 3 (51–75%), and 4 (>75%). The final IHC score was calculated as the product of the staining intensity score and the positive cell proportion score.

### 2.10 Statistical analysis

All statistical analyses and data visualization in this study were conducted using R software (version 4.4.2) and its relevant packages. A two-sided p-value < 0.05 was considered statistically significant (*p < 0.05; **p < 0.01; ***p < 0.001; ****p < 0.0001; ns, not significant).

## 3 Results

### 3.1 Consensus clustering-related analysis

**Figure 1:**
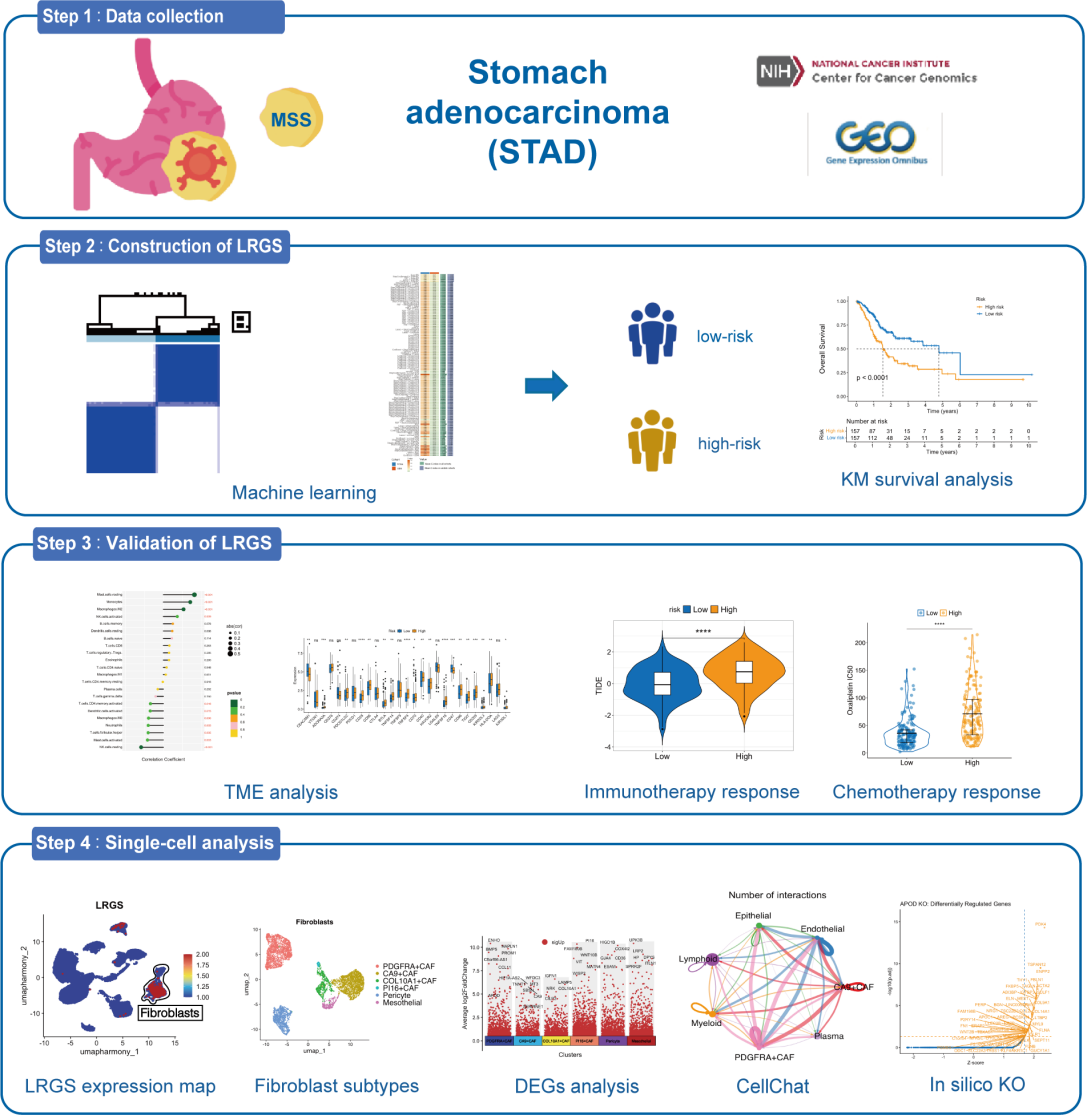
Workflow of the study.

**Figure 2:**
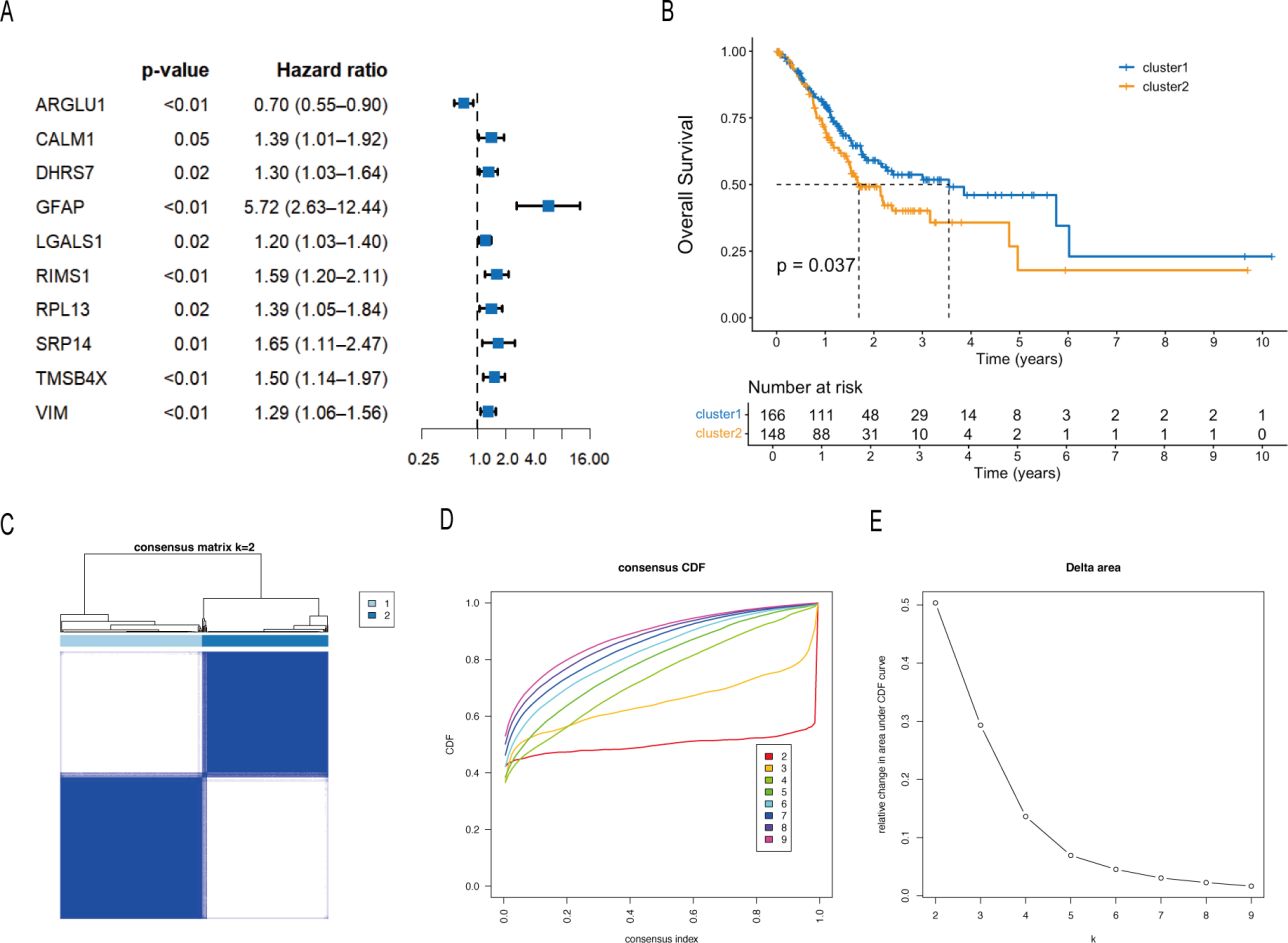
Identification of lactylation-related clusters in MSS GC. **(A)** Univariate Cox regression analysis of lactylation-related genes for prognostic screening. **(B)** Kaplan–Meier survival analysis of the two clusters. **(C)** Consensus matrix heatmap for k = 2. **(D)** CDF curves for k = 2–9. **(E)** Delta area plot illustrating relative changes in CDF area under different k values.

**Figure 3:**
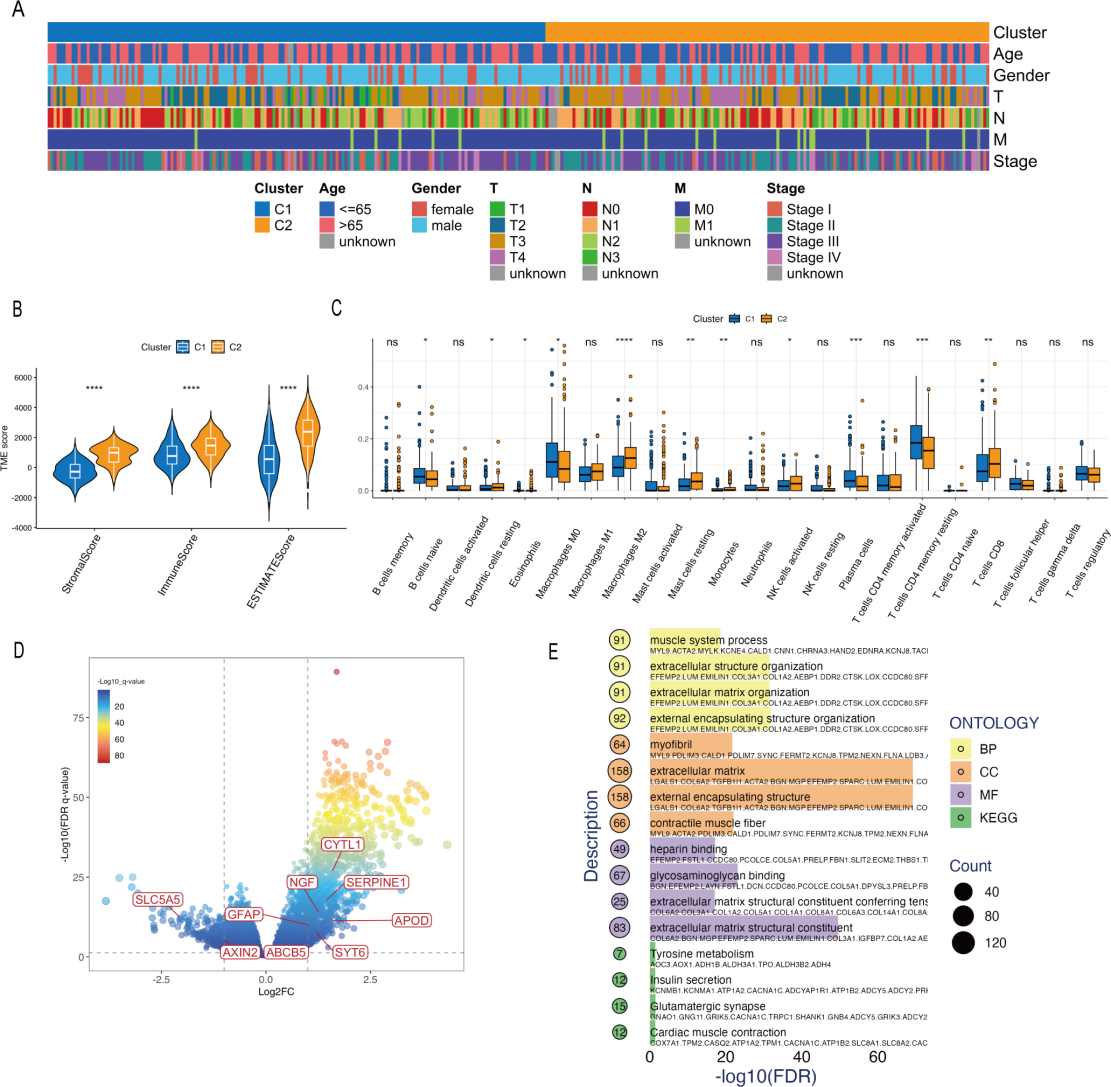
Clinical and TME characteristics of the two clusters in MSS GC. **(A)** Heatmap of clinical characteristics of the two clusters. **(B)** Correlations between the two clusters and TME scores. **(C)** Immune cell infiltration levels of the two clusters. **(D)** Volcano plot of DEGs between the two clusters. **(E)** GO and KEGG enrichment analyses of DEGs. The asterisks indicate statistical significance levels: *p < 0.05, **p < 0.01, ***p < 0.001, ****p < 0.0001; ns, not significant.

The workflow is shown in **Figure 1**. Univariate Cox regression analysis of lactylation-related genes identified 10 genes significantly correlated with patient prognosis (**Figure 2A**). Consensus clustering based on these genes indicated that dividing patients into two clusters was optimal (**Figures 2C–E**). Kaplan–Meier analysis showed that cluster C1 had a significantly better OS than cluster C2 (p = 0.037; **Figure 2B**). Additionally, no significant associations were found between the clusters and clinical parameters (**Figure 3A**).

Given the critical role of the TME, we characterized it using the ESTIMATE algorithm. The ImmuneScore, StromalScore, and ESTIMATEScore were all significantly higher in cluster C2 (**Figure 3B**). Furthermore, CIBERSORT analysis of 22 immune cell types revealed that the infiltration levels of seven immune cells, including resting dendritic cells, eosinophils, M2 macrophages, resting mast cells, monocytes, activated NK cells, and CD8+ T cells, were higher in cluster C2 (**Figure 3C**).

To explore the molecular characteristics underlying these two clusters, we identified DEGs between cluster C1 and C2. A total of 1,228 DEGs were identified, with 256 genes upregulated in C1 and 972 upregulated in C2 (**Figure 3D**). Functional enrichment analysis revealed that genes upregulated in C2 were significantly enriched in extracellular matrix organization, extracellular structure organization, and muscle system process (**Figure 3E**), consistent with its higher stromal scores observed in the TME analysis.

### 3.2 Construction of the LRGS

**Figure 4:**
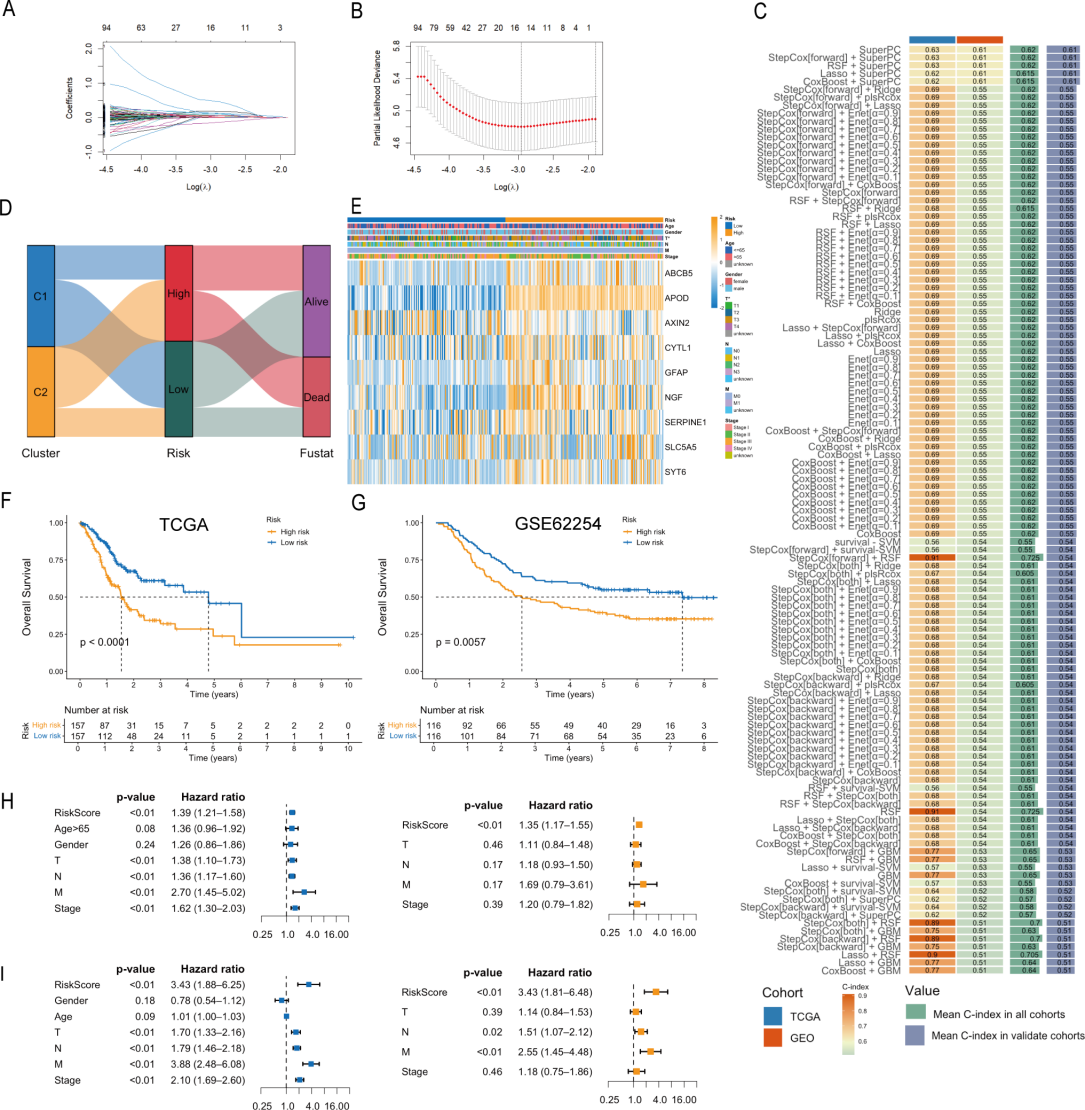
Construction and validation of the LRGS. **(A, B)** LASSO regression analysis of the prognostic genes. **(C)** Construction of LRGS using machine learning algorithms. **(D)** Sankey plot of GC clusters, risk score, and survival status. **(E)** Heatmap of the expression of signature genes and clinicopathological characteristics between the high- and low-risk groups. **(F, G)** Kaplan–Meier survival analysis based on the LRGS in the TCGA **(F)** and GSE62254 **(G)** datasets. **(H, I)** Univariate and multivariate Cox regression analyses of the LRGS in the TCGA **(H)** and GSE62254 **(I)** datasets. The asterisk indicates statistical significance level: *p < 0.05.

We initially employed univariate Cox regression analysis to assess the prognostic significance of DEGs, identifying 380 candidate genes. Subsequently, LASSO regression analysis was performed to further reduce the dimensionality of these 380 genes, culminating in the selection of 15 candidate genes (**Figures 4A, B**). We then integrated 101 combinations derived from 10 machine learning algorithms to independently identify key genes. The concordance indices of these 101 models were evaluated in both the TCGA-STAD dataset and the GSE62254 dataset. The SuperPC model was selected as the optimal model, exhibiting an average C-index of 0.62 (**Figure 4C**). The LRGS encompasses nine genes: *ABCB5, APOD, AXIN2, CYTL1, GFAP, NGF, SERPINE1, SLC5A5*, and *SYT6*.

### 3.3 Validation of the LRGS

Sankey plot analysis revealed that cluster C2 was associated with higher risk scores and poorer prognosis (**Figure 4D**). The heatmap in **Figure 4E** showed the expression patterns of the nine LRGS genes, most of which were upregulated in the high-risk group, alongside significant differences in T stage distribution between the risk groups.

Kaplan–Meier survival analysis demonstrated that patients in the low-risk group had significantly better OS (**Figure 4F**). Furthermore, univariate Cox regression identified LRGS and TNM stage as significant predictors of OS, and multivariate analysis adjusted for age, gender, and TNM stage further confirmed LRGS as an independent prognostic factor (**Figure 4H**). These findings were validated in the GSE62254 dataset (**Figures 4G, I**), indicating the robust predictive ability of the LRGS for survival.

### 3.4 Analysis of TME based on LRGS

**Figure 5:**
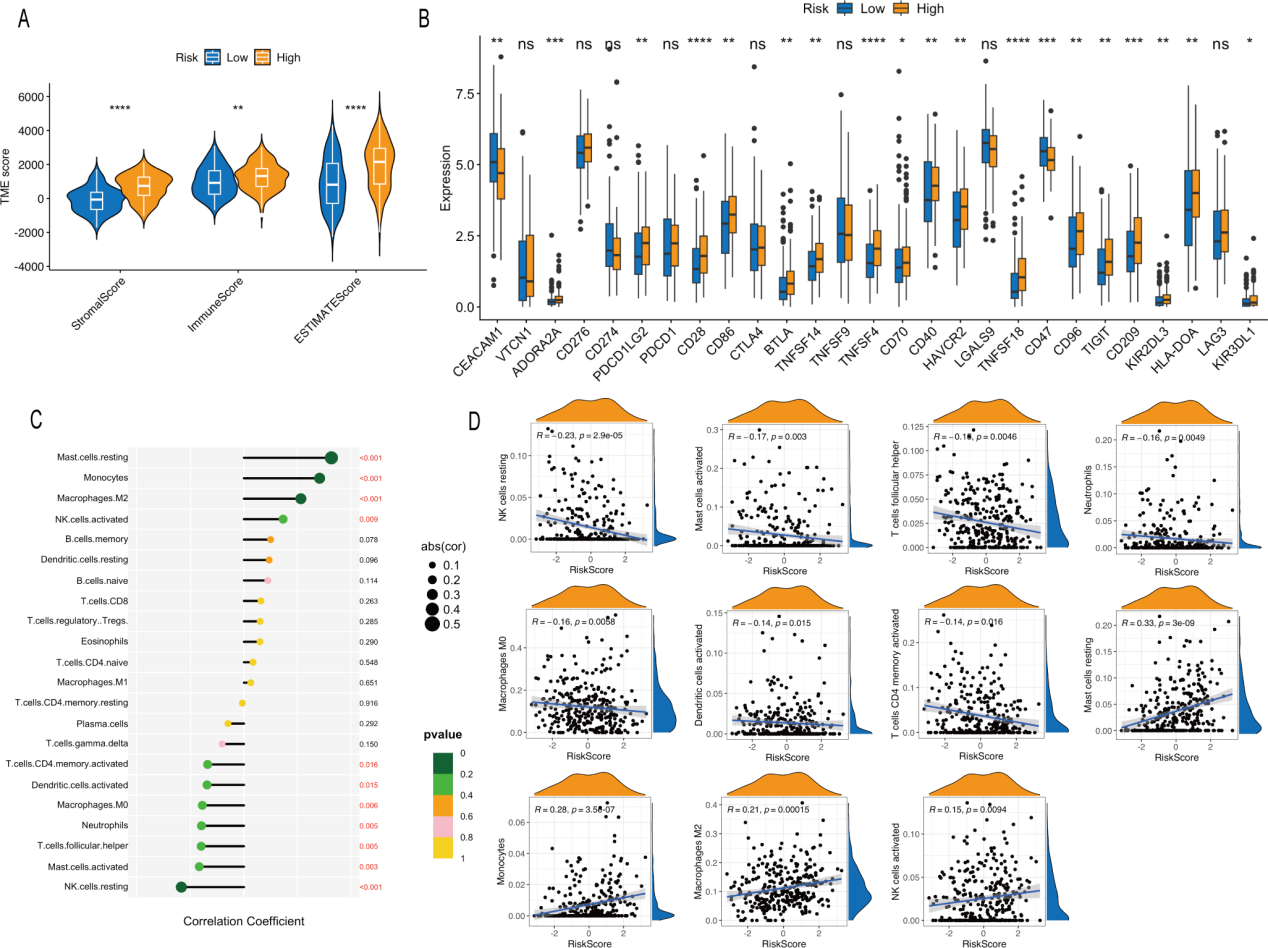
Analysis of TME based on LRGS. **(A)** Correlations between the LRGS and TME scores. **(B)** Expression of immune checkpoint-related genes between the high- and low-risk groups. **(C, D)** Correlations between the LRGS and the infiltration levels of immune cell types. The asterisks indicate statistical significance levels: *p < 0.05, **p < 0.01, ***p < 0.001, ****p < 0.0001; ns, not significant.

To assess the differences in immune cell infiltration between the high- and low-risk groups, we applied the ESTIMATE algorithm and observed elevated ImmuneScore, StromalScore, and ESTIMATEScore in the high-risk group compared with the low-risk group (**Figure 5A**). In addition, immune checkpoint-related genes were differentially expressed between the two groups, with most genes expressed at higher levels in the high-risk group than in the low-risk group (**Figure 5B**). Correlation analysis revealed that LRGS was negatively correlated with CD4+ memory activated T cells, activated dendritic cells, M0 macrophages, neutrophils, T follicular helper cells, activated mast cells, and resting NK cells, and positively correlated with resting mast cells, monocytes, M2 macrophages, and activated NK cells (**Figures 5C, D**).

### 3.5 Immunotherapy and chemotherapy response prediction based on LRGS

**Figure 6:**
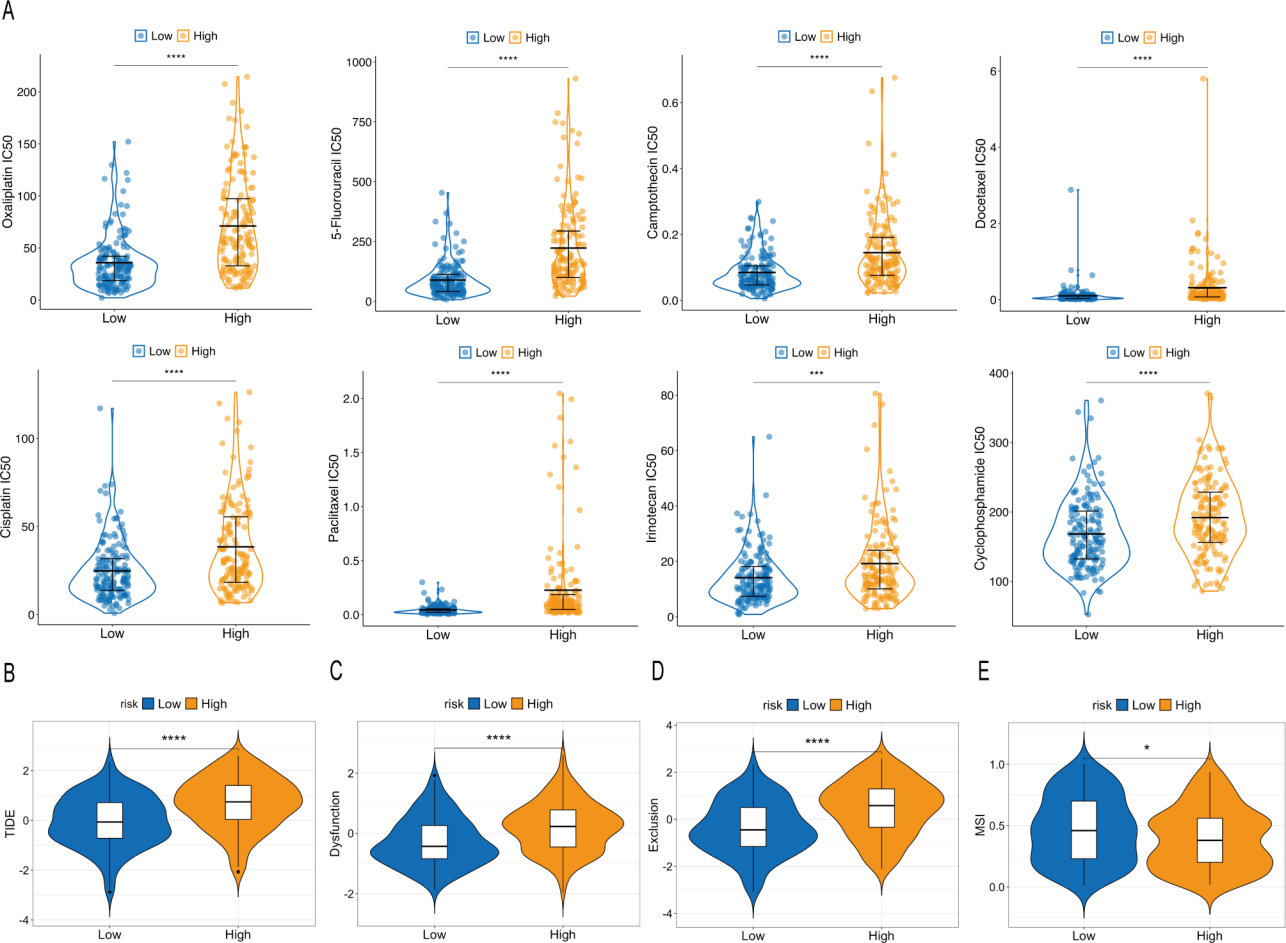
Immunotherapy and chemotherapy response prediction based on LRGS. **(A)** Comparison of IC50 values of chemotherapy between the high- and low-risk groups. **(B)** TIDE scores between the high- and low-risk groups. **(C)** Dysfunction scores between the high- and low-risk groups. **(D)** Exclusion scores between the high- and low-risk groups. **(E)** MSI scores between the high- and low-risk groups. The asterisks indicate statistical significance levels: *p < 0.05, ***p < 0.001, ****p < 0.0001.

We next evaluated the sensitivity of patients in the high- and low-risk groups to chemotherapy commonly used in GC treatment. As shown in **Figure 6A**, compared with the high-risk group, the low-risk group exhibited lower IC50 values for oxaliplatin, camptothecin, 5-fluorouracil, docetaxel, cisplatin, paclitaxel, irinotecan, and cyclophosphamide, suggesting that the low-risk group may derive more benefit from these chemotherapies.

According to TIDE scoring, the high-risk group showed significantly higher TIDE, Dysfunction, and Exclusion scores than the low-risk group (**Figures 6B–D**). In contrast, the low-risk group exhibited a significantly higher MSI score than the high-risk group (**Figure 6E**). Notably, higher TIDE scores have been associated with immune evasion and resistance to immunotherapy in cancers, whereas higher MSI scores typically predict better immunotherapy response. These results suggest that LRGS may help predict response to immunotherapy in MSS GC.

### 3.6 Single-cell RNA sequencing analysis

**Figure 7:**
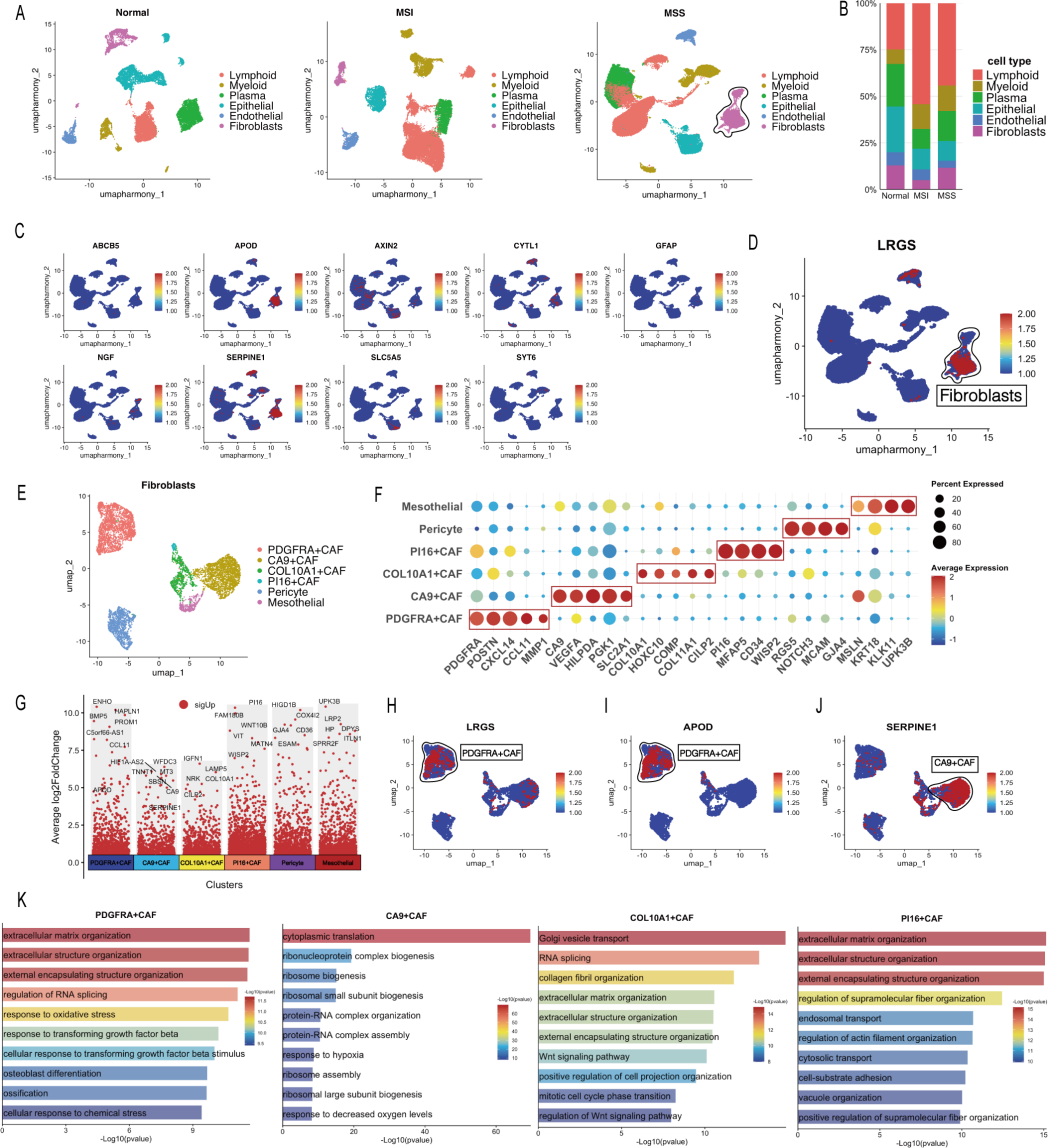
Single-cell atlas of GC and characterization of CAF subtypes. **(A)** UMAP visualization of the major cell types in normal and GC tissues. **(B)** Proportion of major cell types across different sample groups. **(C)** Feature plot of nine LRGS gene expression in GC. **(D)** Feature plot of the distribution of risk score across cell types. **(E)** UMAP visualization of the CAF subtypes. **(F)** Dot plot of the expression of marker genes for CAF subtypes. **(G)** Differential expression analysis highlighting the upregulated genes in CAF subtypes. **(H)** Feature plots of distribution of risk score across CAF subtypes. **(I, J)** Feature plot of *APOD* **(I)** and *SERPINE1* **(J)** expression in CAF subtypes. **(K)** GO enrichment analysis of upregulated genes in CAF subtypes.

Following standardization and analysis using the “Seurat” pipeline, this study included three sample types: normal tissue, MSS GC, and MSI GC. A total of six cell types were identified: epithelial cells, fibroblasts, endothelial cells, lymphocytes, myeloid cells, and plasma cells (**Figure 7A**). Comparative analysis of cellular composition revealed that MSS samples exhibited a lower proportion of lymphocytes but a higher proportion of cancer-associated fibroblasts (CAFs) compared with MSI samples (**Figure 7B**). These findings suggested differences in immune infiltration and stromal components of the TME between these subtypes. We analyzed the expression profiles of nine signature genes at the single-cell level. *APOD* was predominantly expressed in CAFs, whereas *SERPINE1* was highly expressed in both CAFs and endothelial cells (**Figure 7C**). Most of the remaining genes displayed low to moderate expression across the examined cell types. Risk score analysis revealed that, within MSS GC tissues, the CAF population exhibited a higher score compared with other cell types (**Figure 7D**).

Given the significant functional heterogeneity of CAFs in the TME, we focused on this population for further subclustering analysis. Reclustering of the CAF population identified four distinct CAF subtypes, as well as pericytes and mesothelial cells (**Figure 7E**). These CAF subtypes were defined based on their specifically and highly expressed marker genes: PDGFRA+ CAFs, CA9+ CAFs, COL10A1+ CAFs, and PI16+ CAFs (**Figures 7F, G**). The PDGFRA+ CAFs exhibited elevated LRGS scores (**Figure 7H**), and differential expression analysis further revealed that *APOD* was specifically expressed in this subtype (**Figure 7I**). Functional enrichment analysis showed that this subtype was primarily enriched in extracellular matrix-related pathways (**Figure 7K**). In contrast, the CA9+ CAFs exhibited high *SERPINE1* expression (**Figure 7J**) and were functionally linked to pathways such as cytoplasmic translation, ribonucleoprotein complex biogenesis, and hypoxia response (**Figure 7K**). Furthermore, the COL10A1+ CAFs showed enrichment for Golgi vesicle transport and RNA splicing, while the PI16+ CAFs were enriched in pathways related to extracellular matrix organization and regulation of supramolecular fiber organization (**Figure 7K**).

### 3.7 Cell-cell communication analysis

**Figure 8:**
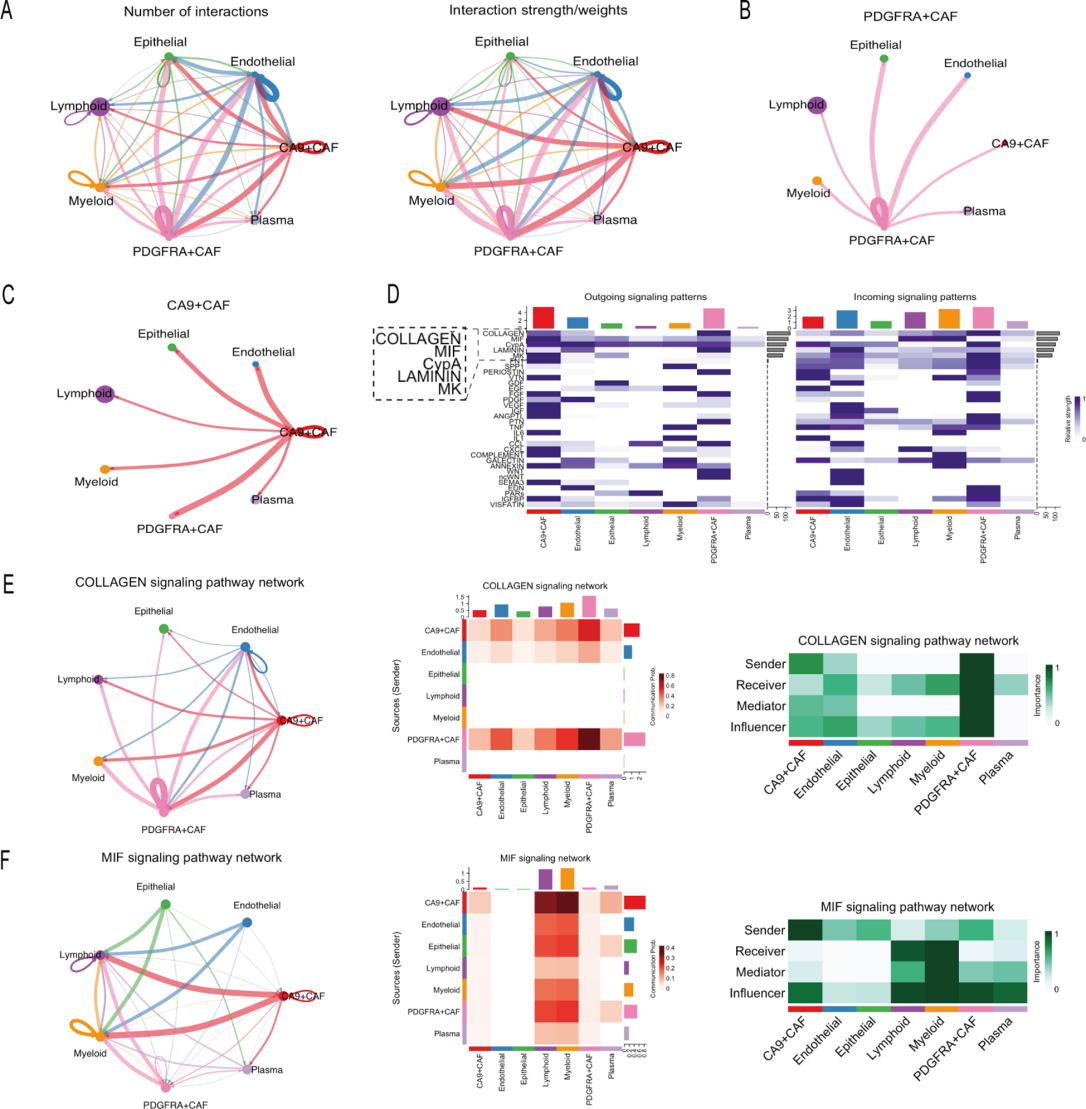
Analysis of intercellular communication in the TME. **(A)** Analysis of the number of interactions and interaction strength in GC. **(B)** Intercellular interactions between PDGFRA+ CAFs and other TME cells. **(C)** Intercellular interactions between CA9+ CAFs and other TME cells. **(D)** Overview of the outgoing signaling and incoming signaling in GC. **(E, F)** Intercellular communication network, interaction strength heatmap, and functional role heatmap of distinct cell types in the COLLAGEN **(E)** and MIF **(F)** signaling pathways.

We analyzed the crosstalk between different cell types in the TME using the CellChat algorithm. The total number of interactions and the interaction strength are shown in **Figure 8A**. Network analysis centered on the two CAF subtypes revealed that CA9+ CAFs exhibited the strongest connection with PDGFRA+ CAFs, while PDGFRA+ CAFs also showed prominent interactions with endothelial and epithelial cells (**Figures 8B, C**).

We analyzed the top two signaling pathway networks and observed distinct functional specialization between the two CAF subtypes (**Figure 8D**). In the COLLAGEN pathway, PDGFRA+ CAFs primarily acted as signal receivers, whereas CA9+ CAFs served as major senders. Notably, PDGFRA+ CAFs exhibited strong connections with myeloid cells, suggesting their involvement in influencing myeloid cells through matrix remodeling (**Figure 8E**). In the MIF pathway, PDGFRA+ CAFs displayed a multifunctional role, exhibiting the highest centrality in receiver, mediator, and influencer dimensions, along with extensive connections to lymphoid and myeloid cells, indicating their active regulation of immune cell function via MIF signaling. In contrast, CA9+ CAFs predominantly functioned as signal senders in the MIF pathway (**Figure 8F**).

### 3.8 In silico KO analysis

**Figure 9:**
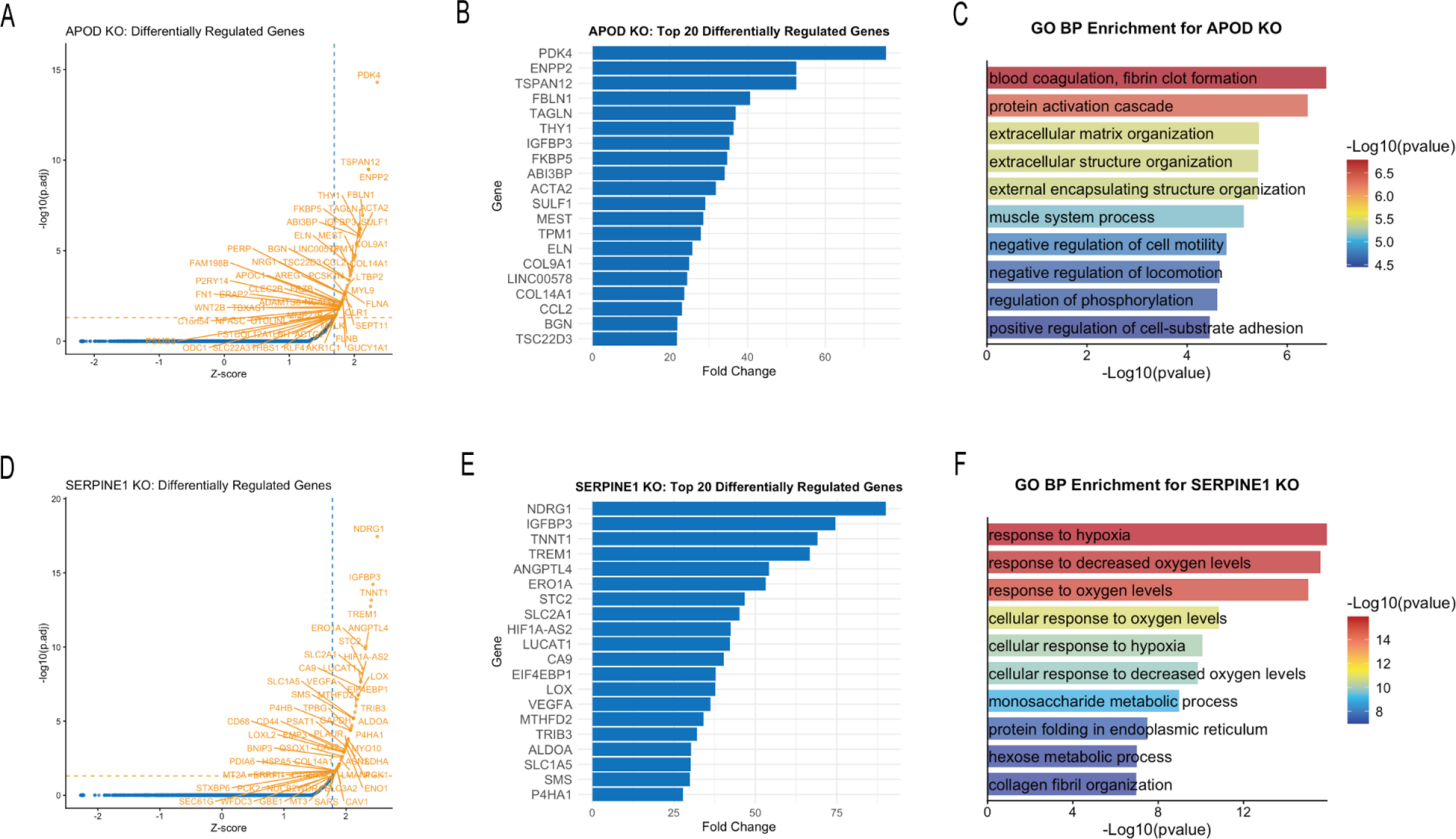
In silico KO analysis of *APOD* and *SERPINE1*. **(A)** Volcano plot of significantly perturbed genes following in silico KO of *APOD*. **(B)** Bar plot of the top 20 perturbed genes following in silico KO of *APOD*. **(C)** GO enrichment analysis of the perturbed genes following in silico KO of *APOD*. **(D)** Volcano plot of significantly perturbed genes following in silico KO of *SERPINE1*. **(E)** Bar plot of the top 20 perturbed genes following in silico KO of *SERPINE1*. **(F)** GO enrichment analysis of the perturbed genes following in silico KO of *SERPINE1*.

In silico KO analysis revealed that *APOD* and *SERPINE1* KO perturbed 57 and 66 downstream genes, respectively (**Figures 9A, B, D, E**). Enrichment analysis showed that the downstream affected genes of *APOD* KO were significantly enriched in pathways related to extracellular matrix organization and regulation of cell motility (**Figure 9C**), whereas *SERPINE1* KO primarily impacted biological processes involved in cellular response to hypoxia and glucose metabolism (**Figure 9F**).

### 3.9 IHC analysis results

**Figure 10:**
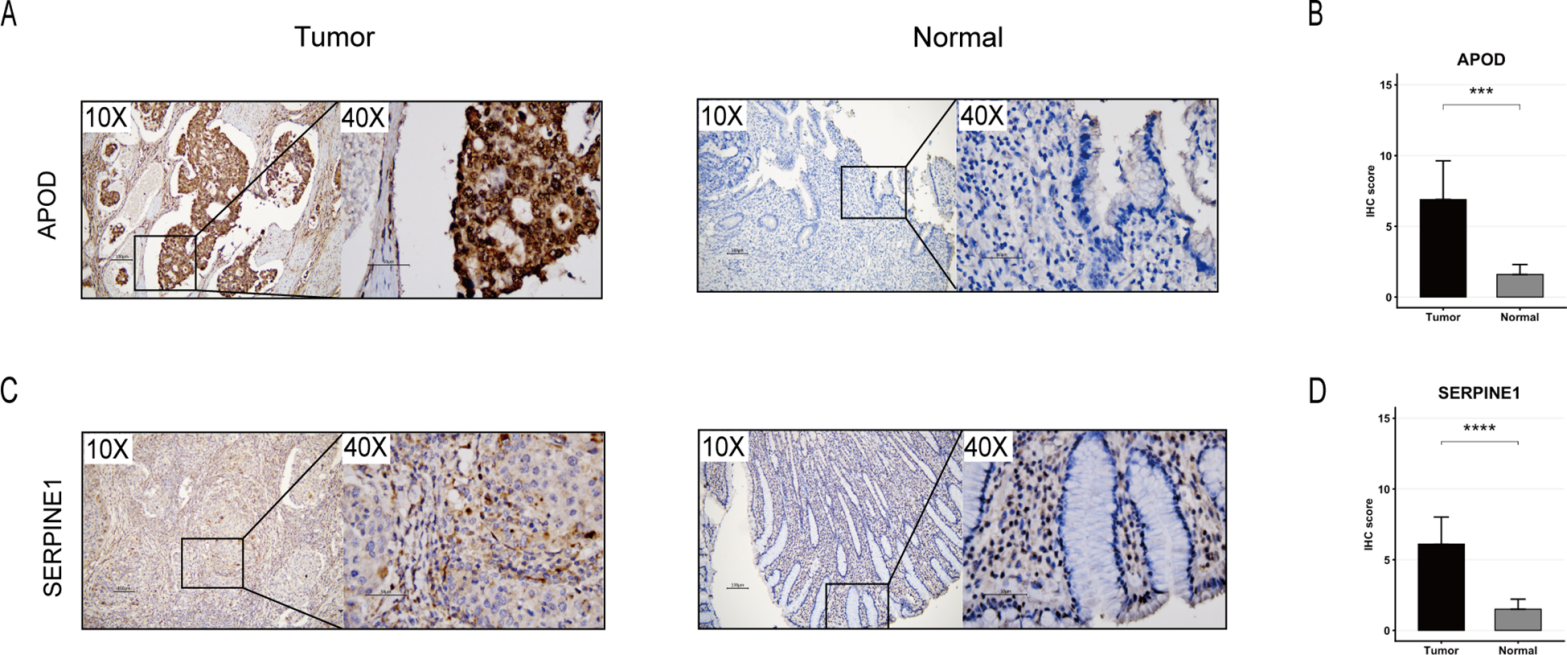
IHC validation of *APOD* and *SERPINE1* expression in MSS GC. **(A)** Representative IHC staining of *APOD* in paired tumor and adjacent normal tissues (magnifications: 10×, 40×). **(B)** Boxplot comparing IHC scores of *APOD* between tumor and normal tissues. **(C)** Representative IHC staining of *SERPINE1* in paired tumor and adjacent normal tissues (magnifications: 10×, 40×). **(D)** Boxplot comparing IHC scores of *SERPINE1* between tumor and normal tissues. The asterisks indicate statistical significance levels: ***p < 0.001, ****p < 0.0001.

To validate the differential expression of *APOD* and *SERPINE1* at the protein level, we performed IHC staining on GC tissues and paired adjacent normal tissues. IHC analysis revealed that positive staining for both *APOD* and *SERPINE1* was predominantly localized in the cytoplasm (**Figures 10A, C**). MSS GC exhibited significantly higher expression of *APOD* and *SERPINE1* than adjacent normal tissues, with statistically significant differences in IHC scores (**Figures 10A–D**). These findings further corroborated our conclusions at the protein level.

## 4 Discussion

MSS GC represents the predominant subtype of GC, and its low response rate to immunotherapy remains a major challenge in clinical management. Significant molecular heterogeneity exists within this subtype, profoundly affecting patient prognosis and treatment sensitivity (19). However, the underlying regulatory networks driving this heterogeneity and immunosuppressive microenvironment formation remain insufficiently understood. Growing evidence indicates that tumor progression is coordinately controlled by a complex interplay of metabolic reprogramming, epigenetic regulation, and post-translational modifications (21–23, 29–31). To address this, our study focused on the role of lactylation-related genes in MSS GC. Through systematic analysis, we developed and validated a nine-gene LRGS. The LRGS not only stably predicted patient outcomes but was also confirmed as an independent prognostic factor in multivariate analysis. More importantly, the risk stratification based on the LRGS was significantly associated with chemotherapy sensitivity and specific immune microenvironment features, suggesting that lactylation may play a key role in regulating treatment response in MSS GC.

We constructed the LRGS, which comprised nine genes including the key regulators *SERPINE1* and *APOD*, along with other genes involved in diverse oncogenic processes such as *NGF, AXIN2*, and *ABCB5* (32–34). Among them, *SERPINE1*, a pivotal fibrinolysis regulator, is frequently overexpressed in cancers and promotes GC progression by inducing epithelial–mesenchymal transition (35, 36). *APOD*, as a key lipid metabolism regulator, is significantly associated with poor prognosis and immunotherapy resistance (37). Collectively, these genes formed the LRGS, which demonstrated robust prognostic power in MSS GC. It exhibited stable performance in the training dataset, effectively stratifying patients into high- and low-risk groups with significantly different OS, and maintained good reproducibility in an independent validation dataset. Multivariate analysis confirmed the LRGS as a significant independent prognostic factor, providing additional predictive value to conventional clinical parameters such as TNM stage.

Based on the validation of the prognostic value of the LRGS, this study further evaluated its potential clinical utility in predicting responses to chemotherapy and immunotherapy. Analysis revealed that the low-risk group exhibited more favorable immune characteristics and increased sensitivity to commonly used chemotherapies such as oxaliplatin and 5-fluorouracil. In contrast, the high-risk group showed elevated TIDE scores, upregulation of multiple immune checkpoints, and polarization of M2 macrophages, which collectively shaped an immunosuppressive microenvironment that drives tumor progression and confers resistance to immunotherapy (38–40). Notably, Cluster C2, which mainly corresponds to this high-risk phenotype, also exhibited increased infiltration of CD8+ T cells and activated NK cells. This apparent paradox can be explained by functional impairment and spatial exclusion of these cytotoxic lymphocytes (40). In this study, the high-risk group presented significantly elevated stromal scores and CAF enrichment. This suggests that CAFs may form dense structural barriers that sequester CD8+ T cells and NK cells in the peritumoral stroma, preventing their infiltration into the tumor core and rendering them ineffective despite their abundance (41–43). Consequently, although the infiltration of CD8+ T cells and NK cells was increased in the high-risk group, spatial exclusion and functional impairment collectively led to failed antitumor immunity and poor prognosis. These findings align with the mechanism by which lactylation modulates the TME. The key gene *SERPINE1* has been confirmed to be closely related to the formation of an immunosuppressive microenvironment, further supporting the predictive value of the LRGS (44–46). In summary, the LRGS serves as a reliable prognostic tool and can guide chemotherapy and immunotherapy decisions for MSS GC patients.

To explore the cellular basis underlying these observations, we further identified four distinct CAF subtypes, which exhibited unique gene expression profiles and were associated with different enriched pathways. Single-cell subcluster analysis further mapped the key genes of LRGS to specific functional CAF subtypes. Specifically, *APOD* was predominantly expressed in the PDGFRA+ CAFs. This subtype was enriched in pathways related to extracellular matrix organization and TGF-β signaling, and exhibited a hybrid phenotype sharing characteristics of both myofibroblasts and inflammatory fibroblasts. This phenotype is consistent with the previously reported MMP1+ myofibroblast population, which contributes to the formation of an immunosuppressive niche and immunotherapy resistance (47). In contrast, *SERPINE1* was specifically expressed in the CA9+ CAFs. This subtype was enriched in pathways related to hypoxia response, ribosome biogenesis, and metabolic reprogramming, functionally resembling the reported metabolic CAFs (48). It may mediate the reverse Warburg effect and undergo metabolic reprogramming to fuel cancer cells, thereby contributing to tumor progression and therapy resistance (49). Cell-cell communication analysis further revealed that these two CAF subtypes exhibited distinct signaling patterns: PDGFRA+ CAFs primarily acted as signal receivers in the COLLAGEN pathway and multifunctional regulators in the MIF pathway, extensively connecting with myeloid and lymphoid cells, whereas CA9+ CAFs predominantly functioned as signal senders. The COLLAGEN pathway has been shown to create a physical barrier that restricts T cell infiltration into tumor nests, thereby contributing to immune exclusion in the TME (50, 51). Meanwhile, MIF signaling may promote M2 macrophage polarization and T cell exhaustion, facilitating an immunosuppressive environment that supports GC progression and immunotherapy resistance (52, 53). These findings provide mechanistic insights into how these CAF subtypes differentially modulate the TME. Furthermore, in silico KO analysis validated that *APOD* and *SERPINE1* are essential for extracellular matrix remodeling and hypoxia adaptation, respectively, directly supporting their functional roles as key drivers within their respective CAF subtypes. Additionally, we identified a CAF subtype characterized by high expression of *PI16, MFAP5*, and *CD34*, whose genetic profile suggests it may represent a fibroblast precursor or progenitor state, consistent with prior reports (47, 54). Finally, COL10A1+ CAFs enriched in extracellular matrix organization and Wnt signaling pathways were also identified. These findings collectively delineate the high heterogeneity and complex functional division of CAFs in MSS GC. In summary, the prognostic power of LRGS likely stems from its key genes precisely identifying the PDGFRA+ CAFs and CA9+ CAFs as effector populations that drive tumor progression.

The main innovation of this study lies in its focus on MSS GC, a cohort known for its poor response to immunotherapy and thus limited treatment options, and the development of the LRGS for precise risk stratification. The LRGS not only provides a new metabolic perspective for understanding tumor heterogeneity and prognostic differences in MSS GC but also serves as a potential guide for formulating personalized treatment strategies. However, several limitations should be acknowledged. First, the IHC validation of the key genes was based on a relatively small sample size, and the correlation between their expression and immune cell infiltration was not analyzed at the protein level. Second, the single-cell RNA sequencing analysis relied on only one public dataset, which may limit its generalizability, and the in silico KO predictions for the key genes were not validated through *in vitro* or *in vivo* functional experiments. Third, the immunotherapy response prediction based on the TIDE algorithm was purely computational and lacked validation using real-world clinical cohorts. These shortcomings highlight important directions for future research.

## 5 Conclusions

This study developed the LRGS based on lactylation-related genes, which effectively enables risk stratification and outcome prediction for patients with MSS GC and shows potential value in predicting treatment response. Critically, single-cell analysis provided a cellular basis for the LRGS by linking its key genes to specific functional CAF subtypes. Future multi-center, prospective clinical studies are warranted to further validate the robustness and applicability of this signature and to elucidate the lactylation-driven regulatory mechanisms of the key genes involved. The signature is expected to provide a valuable basis for developing individualized treatment strategies for distinct risk groups.

## Data Availability

The original contributions presented in the study are included in the article/**Supplementary Material**. Further inquiries can be directed to the corresponding authors.
